# Left Atrial Diverticula Supplied by the Anomalistic Branch of the Right Coronary Artery

**DOI:** 10.7759/cureus.54881

**Published:** 2024-02-25

**Authors:** Hidekazu Takeuchi

**Affiliations:** 1 Internal Medicine (Cardiology), Takeuchi Naika Clinic, Ogachi-Gun, JPN

**Keywords:** lad artery, la diverticula, tee, cardiac ct, la thrombi, pulmonary vein thrombi, pulmonary vein thrombosis

## Abstract

We have reported several cases of pulmonary vein thrombosis in elderly individuals with or without chest pain; pulmonary vein thrombosis is common in aged individuals and should be evaluated further. However, the properties and roles of pulmonary vein thrombi (PVTs) have not been determined. During infection, neutrophil extracellular traps (NETs) are produced to kill pathogens, and arterial thrombi (ATs) are produced in pulmonary veins to prevent pathogens from spreading to all organs. We reported that fine PVTs became larger PVTs and extended to the LA wall.

PVTs can cause acute myocardial infarction (AMI) and ischemic stroke (IS) by releasing larger particles; therefore, the characteristics of PVTs need to be determined to prevent the occurrence of AMI and IS. PVTs can cause several diseases by releasing smaller particles, such as NETs, for which cumulative effects should be determined. PVTs and their effects on human health need to be studied to avoid missing the chances of treating patients with these diseases moderately. We reported that PVTs often extend to the left atrium (LA) and attach to the LA wall; however, the effects of attachment remain unclear.

According to cardiac computed tomography (CT), left atrial diverticula (LADs) reportedly occur in 10%-50% of patients; however, the details of the LAD are unknown. Therefore, we examined the relationships among PVTs, LA thrombi, and LADs using cardiac CT and transesophageal echocardiography (TEE). The patient was a 65-year-old male with hypertension and severe palpitations. He had no history of AMI or IS.

TEE revealed that the LA thrombi were attached to the anterior wall of the right lower pulmonary vein and that they were attached to the anterior wall of the LA. TEE revealed an LAD near the attachment area. Cardiac CT revealed an LAD without thrombi near the attachment area. Sagittal images from a cardiac CT scan revealed that a part of the attachment region in the LA was a dark line, where no blood flow was observed in the LA, and that there seemed to be the LAD on top of the dark line. The anomalistic branch of the right coronary artery (#1) connected around the top of the LAD.

## Introduction

Pulmonary vein thrombosis is common in elderly people with chest pain and should be evaluated [1]. In many patients with pulmonary vein thrombosis, thrombi are observed in several pulmonary veins [2], suggesting that pulmonary vein thrombosis is common in aged patients. Furthermore, we reported several cases of pulmonary vein thrombosis with or without chest pain using cardiac computed tomography (CT), transthoracic echocardiography (TTE), and transesophageal echocardiography (TEE) [1-7], suggesting that pulmonary vein thrombosis is common in aged patients. Respiratory infections generate neutrophil extracellular traps (NETs) to kill pathogens [8], and NETs generate arterial thrombi (ATs) to prevent pathogens from spreading to all organs in pulmonary veins [9]. We have reported that fine pulmonary vein thrombi (PVTs) become long and thick PVTs [3], expand to the LA [2,5,7], and attach to the LA wall [4,6]. However, there are many unclear issues related to PVTs including their traits and roles.

PVTs can cause acute myocardial infarction (AMI) [10], ischemic stroke (IS) [11], and arterial thrombosis [12] by releasing larger particles. Moreover, PVTs can cause several diseases by releasing smaller particles, including NETs and other substances such as DNA and histones that can affect all organs cumulatively. NETs are known to be associated with many diseases, such as acute coronary syndrome [13], heart failure [14], type 2 diabetes mellitus [15], and cancer [16], suggesting that treatment with PVTs may affect these diseases. Therefore, the characteristics of PVTs need to be determined to prevent the occurrence of AMI, IS, systemic thrombosis, and NET-associated diseases.

Left atrial diverticula (LADs) reportedly occur in 10%-50% of patients who undergo cardiac CT [17-19]; however, the details of LAD formation are unknown. Therefore, we investigated the relationships among PVTs, LA thrombi, and LADs.

## Case presentation

The patient was a 65-year-old male with hypertension and severe palpitations. He had no symptoms of chest pain, fever, cough, sputum, or cerebral infarction. The cardiac exam did not reveal an arrhythmia or a heart murmur. The respiratory exam did not reveal decreased breath sounds, lung crackles, or wheezing. His BMI was 23.9 kg/m^2^. A chest roentgenogram revealed no lung cancer or cardiomegaly. No previous treatment with warfarin or direct oral anticoagulants was performed. ECG indicated sinus rhythm, negative T in I, aVL, and V3-V6, and a heart rate of 52 beats/min. The serum D-dimer level was 0.6 μg/mL (normal; <1.0 μg/mL), the activity of protein S was 112% (normal; 74-132%), and the activity of protein C was 105% (normal; 64-135%). The homocysteine level was 5.5 nmol/mL (normal; 5-15 nmol/mL). His brain natriuretic peptide level was 67.8 pg/mL (normal; 0.0~18.4 pg/mL). His C-reactive protein level was 0.05 mg/dL (normal; 0.00~0.19 mg/dL). To further evaluate the thrombi in the LA, pulmonary veins, and LADs, TEE and cardiac CT were performed using 80-slice multidetector computed tomography (80-MDCT) images.

**Figure 1:**
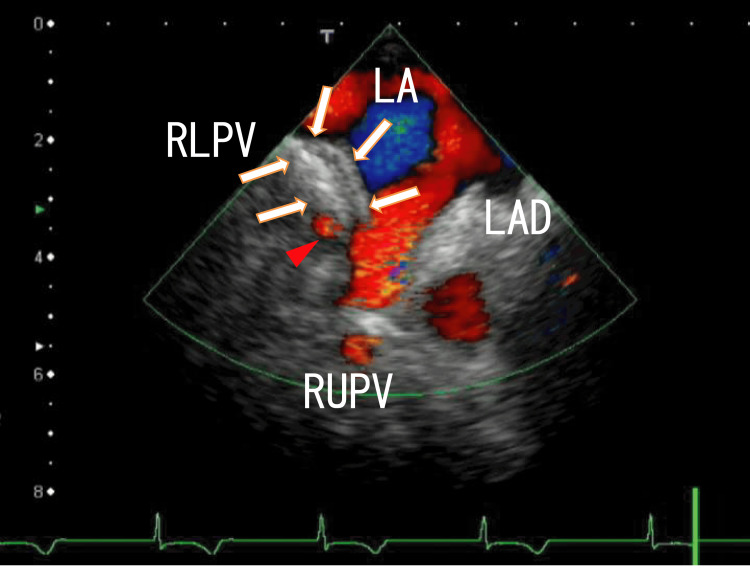
TEE images demonstrating thrombi in the left atrium TEE images demonstrate extended LA thrombi from the RLPV in the LA. The LA thrombi were depicted as white thick line-like shapes that appeared to be connected to the right side of the entrance of the RUPV with a thin line (arrows). The RLPV and RUPV blood flows are shown as red areas. The thrombi included a line-like white area and were linear in shape. There was a small red area between the thrombi and the wall of the left atrium (arrowhead). The border of the thrombi and LA wall was vague and the thrombi and LA wall appeared as a mass. The LAD was depicted on the left side of the LA anterosuperior wall near the entrance of the RUPV. No thrombi were detected in the LAD. TEE, transesophageal echocardiography; LA, left atrium; RLPV, right lower pulmonary vein; RUPV, right upper pulmonary vein; LAD, left atrial diverticulum.

TEE demonstrated the large thrombi in the LA as a mass and included white parts. However, the right end of the thrombi was not clearly visible because of the angle (Figure 1 and Video 1).

**Video 1:** TEE images showing thrombi in the left atrium TEE images demonstrate extended LA thrombi from the RLPV in the LA. The LA thrombi were depicted as white thick line-like shapes that appeared to be connected to the right side of the entrance of the RUPV with a thin line (arrows). The angle of this video was the same as that in Figure 1. The thrombi included a line-like white area and were linear in shape. The blood flow from the RLPV and RUPV are shown as red areas. There was a small red area between the thrombi and the wall of the LA, which appeared at the same time as the flow of the RUPV. The border between the thrombi and the LA wall was vague. The larger parts of the thrombi moved while breathing but did not move with the heartbeats. However, the inner thrombi moved periodically with heartbeats, and the left end of the inner thrombi was connected to the left sidewall at the entrance of the RUPV. The LAD was depicted on the right side of the LA anterior wall near the entrance of the RUPV. The LAD moved with the heartbeats, and there were no thrombi in the LAD. TEE, transesophageal echocardiography; LA, left atrium; RLPV, right lower pulmonary vein; RUPV, transesophageal echocardiography; LAD, left atrial diverticulum.

**Figure 2:**
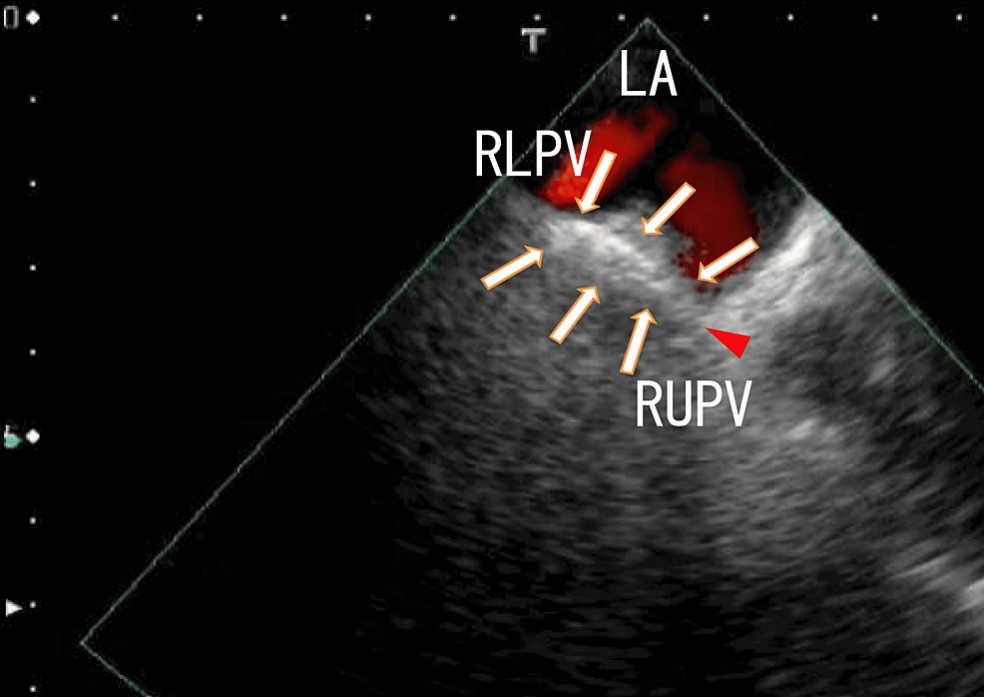
TEE images demonstrating thrombi in the RLPV attached to the LA wall TEE images demonstrate thrombi in the RLPV as white thick line-like shapes that appeared to extend to the LA (arrows) and attached to the anterosuperior wall of the LA (arrowhead). The thrombi included a thick white area and were linear in shape. The blood flow from the RLPV is shown as a bright red area, and the blood flow from the RUPV is shown as a dark red area. TEE, transesophageal echocardiography; LA, left atrium; RLPV, right lower pulmonary vein; RUPV, right upper pulmonary vein.

Therefore, we observed nearer parts of the right lower pulmonary vein (RLPV). The right end of the thrombi seemed to attach to the anterior wall of the RLPV, and the left end of the thrombi was attached to the anterosuperior wall of the LA (Figure 2 and Video 2).

**Video 2:** TEE images demonstrating thrombi in the RLPV attached to the LA wall TEE images demonstrate thrombi in the RLPV as thick white line-like shapes that appeared to extend to the LA. The right end of the thrombi seemed to be attached to the anterior wall of the RLPV, and the left end of the thrombi seemed to be attached to the left anterior wall of the LA near the entrance of the RUPV. The thrombi included a thick white area and were linear in shape, and the white area seemed to have a small white particle with a white shadow. The thrombi moved with the heartbeats. The blood flows from the RLPV and RUPV are shown as red areas. TEE, transesophageal echocardiography; LA, left atrium; RLPV, right lower pulmonary vein; RUPV, right upper pulmonary vein.

**Figure 3:**
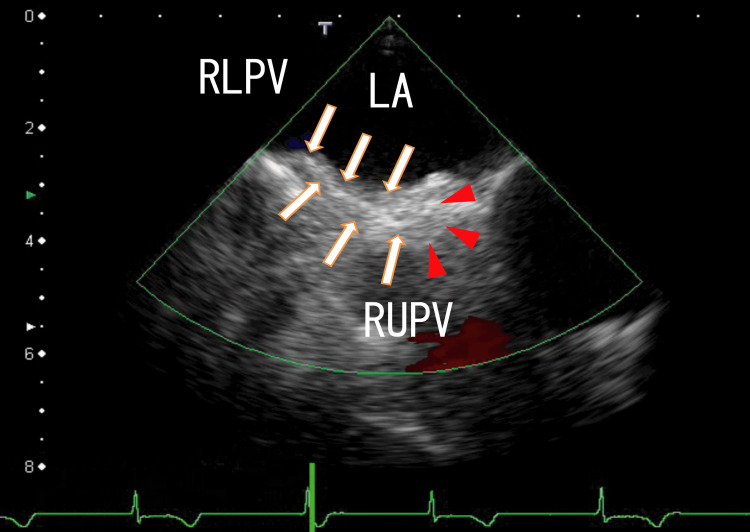
TEE images demonstrating that extended LA thrombi attached to the LA TEE images demonstrate thrombi in the LA as a line-like broad white mass. The inner side of the mass had extended and was attached to the right side of the LA anterosuperior wall near the entrance of the RUPV (arrows). The attached area is dim (arrowheads). The thrombi included a white area and were linearly shaped. The border between the right side of the thrombi and the LA wall was also vague. TEE, transesophageal echocardiography; LA, left atrium; RLPV, right lower pulmonary vein; RUPV, right upper pulmonary vein.

Then, we checked the left side of the LA thrombi. The left end of the thrombi was attached to the anterosuperior wall of the LA (Figure 3 and Video 3).

**Video 3:** TEE images demonstrating that the LA thrombi attached to the LA TEE images demonstrate thrombi in the LA as large white masses. The inner side of the mass was attached to the right side of the LA anterior wall near the entrance of the RUPV. The area of attachment was dim, and the border between the LA thrombi and the LA wall was vague. The thrombi included a white area and were linearly shaped. Around the attachment area, white thin line-like thrombi without shadows appeared at the end of the video. TEE, transesophageal echocardiography; LA, left atrium; RUPV, right upper pulmonary vein.

**Figure 4:**
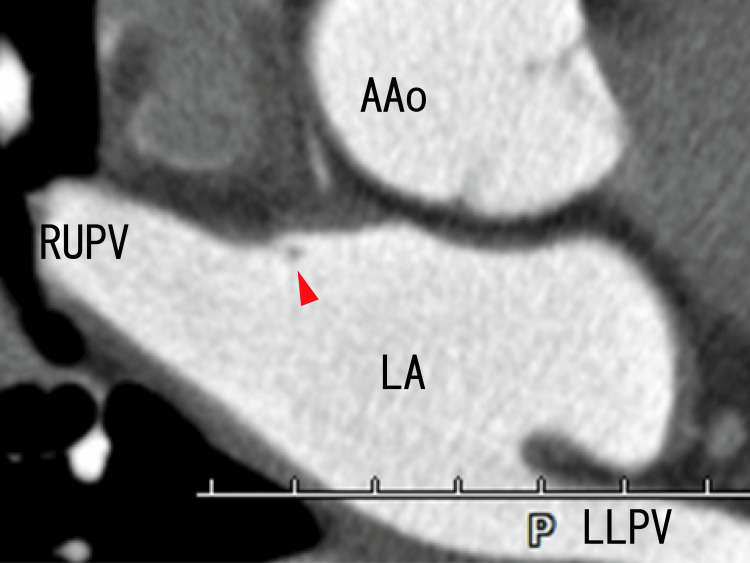
Axial images from an 80-MDCT scan revealing the attachment point in the LA Axial images from an 80-MDCT scan reveale the RUPV, LLPV, and LA. There were no images of thrombi in the LA. There was a dark spot near the anterior wall of the LA (arrowhead), suggesting that both the LA thrombi and the anterior wall of the LA were attached there. The dark spot and the anterior wall of the LA seemed to be connected by a dim thin line. 80-MDCT, 80-slice multidetector computed tomography; AAo, ascending aorta; LA, left atrium; LLPV, left lower pulmonary vein; RUPV, right upper pulmonary vein.

Axial cardiac CT demonstrated no thrombi in the LA; however, there was a dark spot on the anterosuperior wall of the LA, which should be an attachment area (Figure 4).

**Figure 5:**
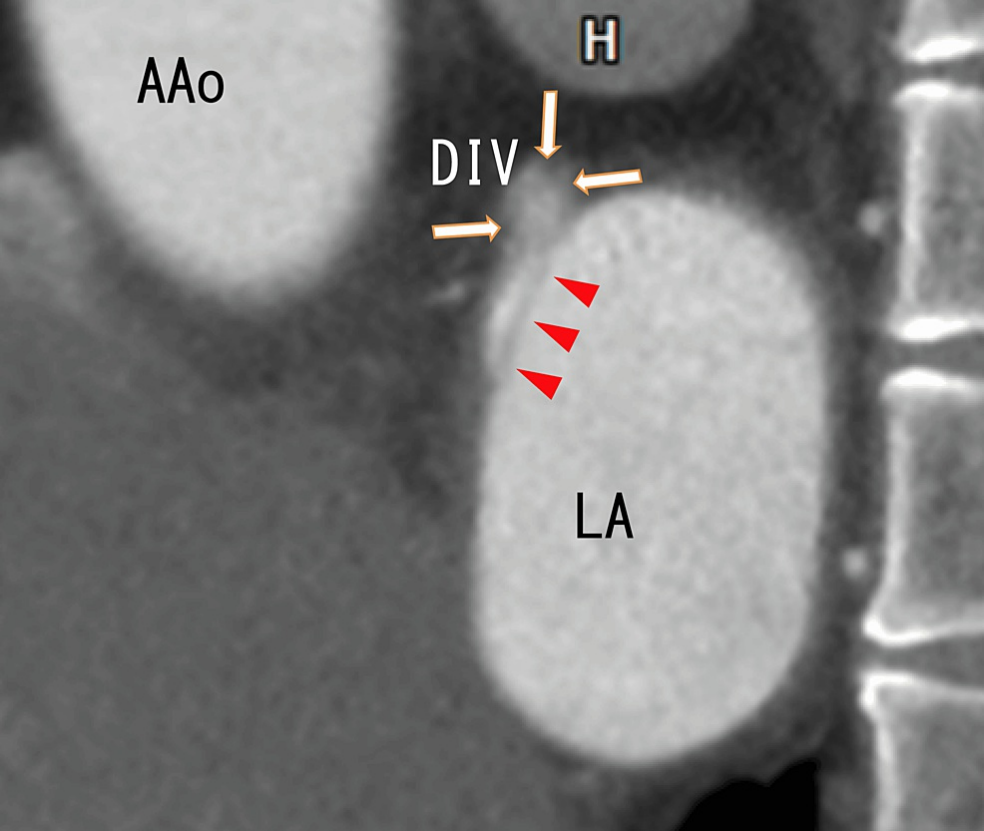
Sagittal images from an 80-MDCT scan revealing the attachment region in the LA and LAD Sagittal images from an 80-MDCT scan reveal an LAD and LA. There were no images of thrombi in the LA. There was a dark line near the anterosuperior wall of the LA (arrowheads), suggesting that the LA thrombi were attached to the anterosuperior wall of the LA. At the top of the line, there was an LAD, the border of which was vaguely depicted. 80-MDCT, 80-slice multidetector computed tomography; AAo, ascending aorta; LA, left atrium; DIV, diverticulum.

Sagittal cardiac CT demonstrated no thrombi in the LA; however, there was a dark line on the anterosuperior wall of the LA, which should be the attachment area. Moreover, the LA diverticulum (LAD) was positioned at the top of the dark line (Figure 5).

**Figure 6:**
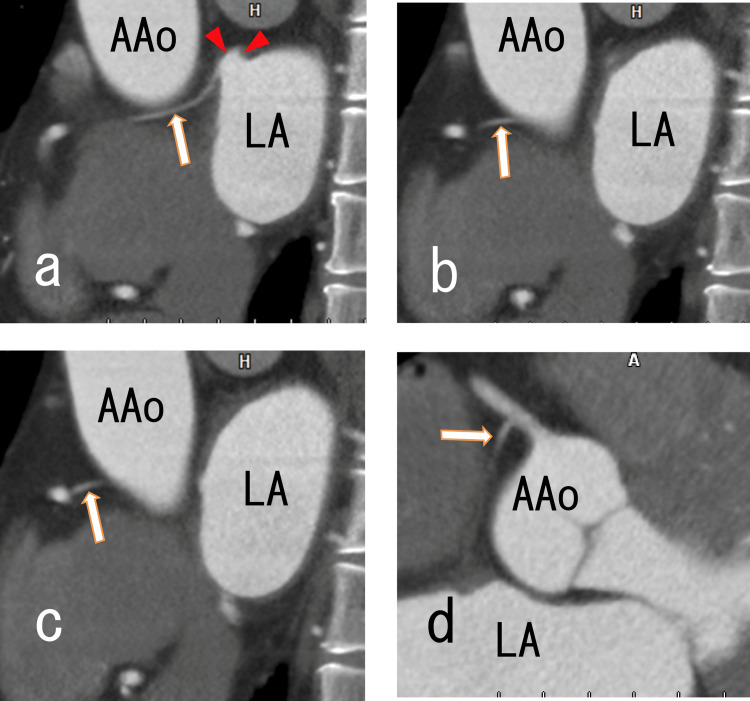
Sagittal and axial images from an 80-MDCT scan revealing a branch of the RCA (#1) that was connected to the LAD Sagittal and axial images from an 80-MDCT scan revealed a branch of the RCA (#1) that connected to the LAD. a. Sagittal images revealing a thin branch (arrow) connected near the top area of the LAD (arrowheads). b. Sagittal images revealing the thin branch (arrow). c. Sagittal images revealing that the thin branch was connected to the RCA (#1) (arrow). d. Axial images revealing the thin branch of the RCA (#1) (arrow). 80-MDCT, 80-slice multidetector computed tomography; RCA, right coronary artery; LAD, left atrial diverticulum; AAo, ascending aorta; LA, left atrium.

Sagittal and axial cardiac CT demonstrated that the branch of the right coronary artery (RCA) (#1) reached near the top of the LAD (Figure 6).

## Discussion

To our knowledge, this is the first report showing that the branch of the RCA (#1) connects near the top of the LAD. The LAD was present near the attachment area; however, the LAD was smaller than the attachment area. The part of the LAD formation area near the LA thrombus attachment area might be the place where the branch of the RCA had reached.

To our knowledge, this is the first report of the use of TEE to reveal thrombi in the LA that are attached to the anterior wall of the proximal RLPV and the left side of the LA wall near the entrance of the right upper pulmonary vein. However, these LA thrombi could not be detected using cardiac CT [6,7]. Previously, we reported thin short thrombi attached to the wall of the left upper pulmonary vein using cardiac CT [20]. The previous thrombi extended to the LA at the free end, but the relationships between thrombi and other tissues are unknown. In the current case, the thrombi attached to the anterior wall of the RLPV appeared to play a role in supporting the LA thrombi. Previously, we reported that PVTs started as small thrombi at the distal pulmonary vein that should be ATs formed based on NETs, which became larger and extended to the LA wall [3,6]. The present case might reveal PVTs that started from the proximal pulmonary vein.

This is the first report showing the movement of the LAD using TEE. Our previous reports revealed LAD thrombi and associated LA thrombi in the upper parts of the LA, which were detected via cardiac CT [6]. However, in the present case, there were no LAD thrombi in or around the LAD, as assessed using TEE and cardiac CT.

In the present case, the right end of the LA thrombi was attached to the anterior wall of the proximal RLPV, and the left end was widely attached to the anterior wall of the LA. The initiation of LA thrombi may have occurred either in the proximal RLPV or at the attachment areas. In any case, NETs may be involved in thrombus formation; however, NETs do not prevent pathogenesis.

Thrombi in the LA may be associated with LAD formation, branch formation, and other LA remodeling. In the attachment area, no or poor blood flow was suspected, indicating that there was no or little oxygen or nutrition. Therefore, the left atrial wall did not actively move. Moreover, cells in the attachment area might lose normal cell function and change their cell structures. The cells might secrete signals to induce branch formation to obtain a blood supply. To come to conclusions, further research is needed.

## Conclusions

The thrombi in the LA were attached to the anterior wall of the proximal RLPV and anterior wall of the LA, and the LAD was near the attachment area on the anterosuperior wall of the LA. The anomalistic branch of the RCA (#1) was connected to the LAD, and the LAD had no thrombi.
